# Annotations

**Published:** 1904-12-10

**Authors:** 


### Annotations.

### Emergency Receiving House, or First Aid Station?

On Thursday next a very important question will be decided at a special general meeting of the trustees of Manchester Royal Infirmary. The Board of Management propose that, in view of the removal of the infirmary to Stanley Grove, accommodation for the treatment of medical and surgical casualties and dispensary out-patients shall be provided near the Piccadilly site. It will be remembered that two years and a half ago the opposition to the removal of the infirmary was largely based upon the argument that the city would be left without prompt and efficient help in cases of emergency, and that it was overcome by an undertaking to meet the objection. The manner in which it is now proposed to redeem the pledge has revived the embers of an almost extinguished controversy. The Board of management are accused of not having kept faith with those who trusted them and of offering a First Aid station instead of the Emergency Receiving House which, it was understood, was in contemplation. In particular, the absence of beds in the scheme has provoked indignation, and it is urged that without a perfect system of ambulance motors, which at present is non-existent, dying and badly injured persons will have to run the grave risks of further removal after first treatment. Only twelve beds are suggested, six for men and six for women and children. The reply of the managers is that even twelve beds would be setting up a separate hospital, entailing many additional things and great additional expense. This, no doubt, is to some extent true ; yet it appears to us that if a question of confidence is involved, the cost of running a few beds ought to be as dust in the balance. The managers also contend that a Receiving House is unnecessary, but its advocates ask for a practical test, and plead that if it be found that beds are not wanted in the neighbourhood of Piccadilly, they can easily be closed and the original scheme be reverted to. The fact that ever since the erection of the Poor Law Hospital at Crumpsall, a quarter of a century ago, the Manchester Board of Guardians have maintained receiving wards with beds in New Bridge Street, and found them essential in the interests of humanity, is yet more eloquent than words. It would certainly be a pity if, the need of a Receiving House being so clearly demonstrated, the supply of it devolved upon other people than the board of an institution which has for so many years been, justly enough, the pride of Manchester, and enjoyed its liberal support.

### The Therapeutical Society.

The issue of a volume of "Transactions" affords substantial evidence of the value of the work which is being accomplished by the recently established Therapeutical Society. The several papers, in accordance with the constitution of the society, deal with such questions as the composition and active principles of drugs, with the practical application of remedies in the treatment of disease, and with physical, electrical, and other therapeutic methods. Clinical and pathological details occupy a subordinate position and are only introduced in so far as they serve to illustrate or justify the propositions which belong to therapeutics. It is not necessary to depreciate other societies in order rightly to appraise the motive and methods of the new organisation. The culture of clinical and pathological knowledge is an interest which lies at the very basis of the medical art and without its adequate prosecution stagnation is inevitable. All the same it is well to be reminded that such knowledge exists not as an end in itself but in order to render practical and fruitful the treatment of disease. It is to this end that all the branches of the medical curriculum exist, and interesting as many of them are in themselves, those who specially cultivate and teach them ought ever to remember that to the practitioner they are mainly important because they form the necessary basis for the practice of therapeutics. Hence there is abundant justification for the establishment of a therapeutical society, and indeed it may fitly be said no society can claim a more secure defence. Judging by the work already accomplished, and by the programme for the current session, there is no lack of material for discussion, and the results must without question be widely spread.

### The Five Years' Curriculum.

When in 1893 the General Medical Council determined to extend the medical curriculum to five years there was a general expectation that the fifth year would be devoted solely to practical and clinical work. Whatever reading may now be given to the exact terms of the Council's decision, the understanding at the time was that systematic lectures should not invade the final year of medical study. It is beyond question that the scheme framed with this object has miscarried. The earlier part of the curriculum has become more detailed and more burdensome, with the result that in the sense of practical training for his life's work the student is no better off than was the case under the four years' term. In a letter written by Sir Wm. Gairdner the responsibility for this state of matters is attached to the overgrowth of the scientific subjects, and more particularly to physiology, and a general confirmation is given to this opinion by the reports recently presented to the General Medical Council on the final examinations of the Scottish Universities. The position in all truth is a most serious and disappointing one. In some quarters there is already a suggestion that the only solution is to be found in the addition of yet another year to the compulsory curriculum. But even were this mechanical plan approved, there is no reason to believe that it would be efficacious. The tactics which have nullified the value of a fifth year would have much the same effect on a sixth. What is needed is an adjustment of the several subjects included in the curriculum in relation to the end for which the curriculum exists—namely, the training of the medical practitioner. Dr. Lindsay Steven has done good service in urging this question on the attention of the General Medical Council, and it is to be hoped that the Education Committee to which it has been referred will make a searching inquiry. The very basis of medical efficiency is concerned in the matter, for under the present practice clinical and practical work is being sacrificed to the claims of abstract science.

